# Silicon Dioxide Nanoparticles Induce Innate Immune Responses and Activate Antioxidant Machinery in Wheat Against *Rhizoctonia solani*

**DOI:** 10.3390/plants10122758

**Published:** 2021-12-14

**Authors:** Abdelrazek S. Abdelrhim, Yasser S. A. Mazrou, Yasser Nehela, Osama O. Atallah, Ranya M. El-Ashmony, Mona F. A. Dawood

**Affiliations:** 1 Department of Plant Pathology, Faculty of Agriculture, Minia University, El-Minya 61512, Egypt; Abdelrazek.sharawy@mu.edu.eg (A.S.A.); ranya.elashmoni@mu.edu.eg (R.M.E.-A.); 2 Business Administration Department, Community College, King Khalid University, Guraiger, Abha 62529, Saudi Arabia; ymazrou@kku.edu.sa or; 3 Department of Agriculture Economic, Faculty of Agriculture, Tanta University, Tanta 31527, Egypt; 4 Department of Agricultural Botany, Faculty of Agriculture, Tanta University, Tanta 31511, Egypt; 5 Citrus Research and Education Center, Department of Plant Pathology, University of Florida, 700 Experiment Station Rd., Lake Alfred, FL 33850, USA; 6 Department of Plant Pathology, Zagazig University, Zagazig 44519, Egypt; osamaoatall1h@ufl.edu; 7 Botany and Microbiology Department, Faculty of Science, Assiut University, Assiut 71516, Egypt; mo_fa87@aun.edu.eg

**Keywords:** *Rhizoctonia solani*, wheat, nanoparticles, silicon dioxide, root rot, damping-off, ROS, antioxidant

## Abstract

The phytopathogenic basidiomycetous fungus, *Rhizoctonia solani*, has a wide range of host plants including members of the family Poaceae, causing damping-off and root rot diseases. In this study, we biosynthesized spherical-shaped silicon dioxide nanoparticles (SiO_2_ NPs; sized between 9.92 and 19.8 nm) using saffron extract and introduced them as a potential alternative therapeutic solution to protect wheat seedlings against *R. solani*. SiO_2_ NPs showed strong dose-dependent fungistatic activity on *R. solani*, and significantly reduced mycelial radial growth (up to 100% growth reduction), mycelium fresh and dry weight, and pre-, post-emergence damping-off, and root rot severities. Moreover, the impact of SiO_2_ NPs on the growth of wheat seedlings and their potential mechanism (s) for disease suppression was deciphered. SiO_2_ NPs application also improved the germination, vegetative growth, and vigor indexes of infected wheat seedlings which indicates no phytotoxicity on treated wheat seedlings. Moreover, SiO_2_ NPs enhanced the content of the photosynthetic pigments (chlorophylls and carotenoids), induced the accumulation of defense-related compounds (particularly salicylic acid), and alleviated the oxidative stress via stimulation of both enzymatic (POD, SOD, APX, CAT, and PPO) and non-enzymatic (phenolics and flavonoids) antioxidant defense machinery. Collectively, our findings demonstrated the potential therapeutic role of SiO_2_ NPs against *R. solani* infection via the simultaneous activation of a multilayered defense system to suppress the pathogen, neutralize the destructive effect of ROS, lipid peroxidation, and methylglyoxal, and maintain their homeostasis within *R. solani*-infected plants.

## 1. Introduction

Wheat (*Triticum aestivum* L.) is an important cereal crop worldwide, which is considered stable food for about one-third of the world population. Wheat plays an important role in total cereal production and global food security [1]. It comes after rice and corn with respect to stable food for most of the world population. *Rhizoctonia spp.* is among various fungal pathogens that cause wheat diseases and affect wheat [2,3]. Two anamorph species of genus *Rhizoctonia*, which causes root disease on wheat and negatively affects its yield, are widely recognized and studied. These include *Rhizoctonia solani* and *Rhizoctonia cerealis*, which are common pathogens, attacking various crops including wheat [4]. *R. solani* Kühn (teleomorph = *Thanatephorus cucumeris* Donk) is a plant pathogenic fungus that causes serious damage and yield losses to wheat plants during the growing season. 

*R. solani* causes what is called bare patch or purple patch which appeared as localized patchy, stunted areas in the field [5,6]. Different countries, such as the Pacific Northwest United States, Australia, Asia, and parts of Europe considered *Rhizoctonia* root rot as a major problem [7,8,9]. The yield losses resulting from *R. solani* averaged up to 20% yearly of wheat crops worldwide [10]. 

Unfortunately, the inoculum of soilborne pathogens that cause root diseases of grown plants in general and wheat and barley specifically often survives longer in the soil as it remains undisturbed and covered within crop residue [11]. Therefore, various management strategies are required to achieve integrated management against damping-off and root rot causal pathogens. The impact of applying conventional fungicides to control *Rhizoctonia* species is vague and not considerable [12,13]. Thus, finding an effective material, which has fungicidal activity and a positive impact on plant growth is urgent. 

Nanotechnology is an important tool of modern science that has contributed to every sector of life. Nanoparticles are particles having a size range between 1 and 100 nm [14,15] and have physicochemical properties which differ from the bulk materials [16]. Nanoparticles may help to improve nutrients, could be used as growth stimulators and as plant protection products. Many studies have confirmed the ability of nanomaterials to improve seed germination and seedling early growth [17,18,19]. A positive effect of different nanoparticles, i.e., titanium dioxide, zinc oxide, nickel, and chitosan on the growth of wheat seedlings were reported [20,21]. Among all known microelements, silicon (Si) comes after oxygen with respect to its abundance, it is the most important mineral element in the soil, it is also recognized as a beneficial nutrient that enhances seed germination, improves and develops plant growth, which leads to increased crop production [19,22]. In addition to plant growth improvement, silicon stimulates the resistance mechanisms of plants against biotic [23,24] and abiotic stress [18,19]. Si is known to suppress many plant diseases, such as bacterial blight, brown spot, grain discoloration, leaf scald, leaf and panicle blast, stem rot, and sheath blight in rice, as well as powdery mildew in wheat and cucumber [25]. In wheat, silicon achieved a significant reduction of many fungal diseases, such as powdery mildew caused by *Blumeria graminis* f. sp. *graminis* [23,26], Septoria leaf blotch [27], leaf blast caused by *Pyricularia oryzae* [28], leaf rust caused by *Puccinia triticina* and yellow spot caused by *Drechslera tritici-repentis* [24], eyespot caused by *Oculimacula yallundae* [29], and spot blotch *Bipolaris sorokiniana* [30]. Therefore, increasing the efficacy of silicon by reducing its particle size to the nano level using a safe synthesizing technique could have a positive influence on its performance in improving plant growth, resistance, and suppressing plant pathogenic fungi. 

In this study, we biosynthesized silicon nanoparticles with unique properties using saffron extract, and we tested the impact of those particles at different concentrations on the growth of wheat seedlings and the severity of wheat root rot disease caused by *R. solani*. The effect of SiO_2_ NPs on the linear growth of *R. solani* was examined as well. In addition, the effect of SiO_2_ NPs on the resistance-related enzymes in wheat seedlings was also evaluated.

## 2. Results

### 2.1. Characterization of SiO_2_ NPs 

**Figure 1:**
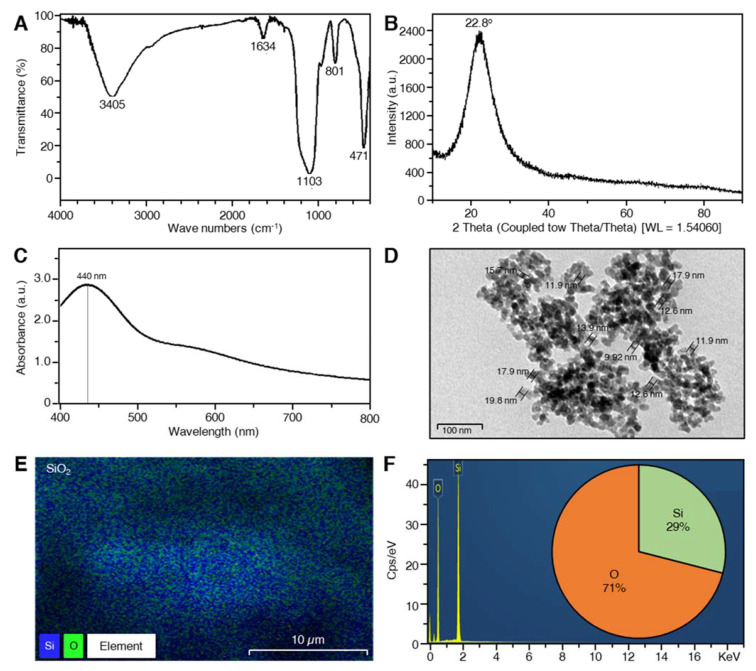
Structural and compositional characterization of silicon dioxide nanoparticles (SiO_2_ NPs). (**A**) FTIR spectrum, (**B**) X-ray diffraction patterns, (**C**) UV-Vis spectrum, (**D**) Bright-field TEM image, (**E**) energy-dispersive X-ray spectroscopy (EDS) elemental mapping of SiO_2_, and (**F**) Energy dispersion spectrum (EDS) and its associated percentage of each element.

The FTIR spectra of SiO_2_ NPs synthesized using saffron stigmas extract showed different bands at 3405, 1634, 1103, 801, and 471 cm^−1^ (Figure 1A), which correspond to O-H stretch hydrogen-bonded, C=C stretch, C-O stretch, and the symmetric vibration of Si atoms, respectively. The XRD spectrum of SiO_2_ NPs expressed broadband with reflection at 2θ = 22.8^o^ and no additional peaks were observed by the XRD spectrum (Figure 1B). The presence of SiO_2_ NPs was confirmed by exhibiting a peak at approximately 440 nm in UV-Vis spectra (Figure 1C). The nanoparticles size ranged from 9.92 to 19.8 nm as obtained from TEM images (Figure 1D). Obtained NPs were spherically shaped, which was detected by using TEM. Data from the energy dispersion spectrum (EDS) of Si Kα1 (Figure S1A), O Kα1 (Figure S1B), and SiO_2_ (Figure 1E) showed that SiO_2_ NPs contained almost 2:1 oxygen and silicon, respectively (71% O and 29% Si; Figure 1F). 

### 2.2. Responses of Wheat Cultivars to R. solani

**Figure 2:**
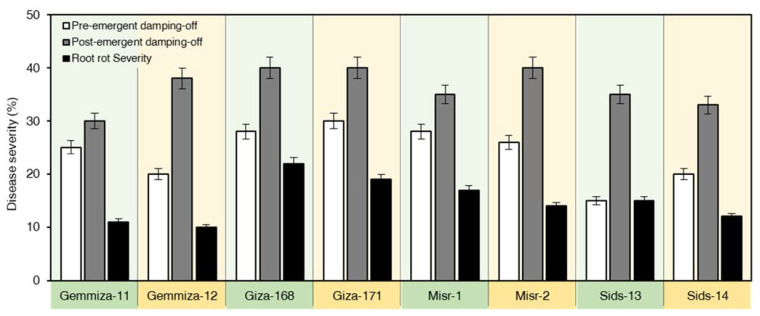
Pathogenicity of *R. solani* against eight Egyptian bread wheat cultivars. The experiment was repeated twice independently. Data presented are means ± standard deviation (mean ± SD) of five biological replicates.

The tested wheat cultivars showed variable responses to *R. solani* infection (Figure 2). The tested isolate expressed symptoms of pre-, post-emergence damping-off and root rot on all wheat cultivars. The highest pre-emergence damping-off values were observed on wheat cvs. Giza-171 (30%), Giza-168 and Misr-1 (28%). However, a low percentage of pre-emergence damping-off was observed on Sids-13 (15%). The percentages of post-emergence damping-off varied from 33 to 40%. Giza-168, Giza-171, and Misr-2 were the most susceptible, which showed 40% post-emergence damping-off, while the lowest post-emergence damping-off was noticed on Gemmeiza-11 (30%). *R. solani* was also able to cause root rot on all wheat cultivars ranging from 10 to 22%. Giza-168 has exhibited the highest root rot (22%), while the lowest root rot (10%) was recorded on Gemmeiza-12 (Figure 2). Additionally, wheat cultivars were estimated to have root rot symptoms caused by *R. solani*. Giza-168 was the most susceptible wheat cultivar showing 22% root rot. Moreover, Giza-171 and Misr-1 showed 19 and 17% root rot, respectively. However, Gemmeiza-11, Gemmeiza-12, and Sids-14 were the most resistant cultivars as they expressed the lowest percentages of root rot and recorded 11, 10 and 12%, respectively (Figure 2).

### 2.3. In Vitro Antifungal Activity of SiO_2_ NPs on R. solani

**Figure 3:**
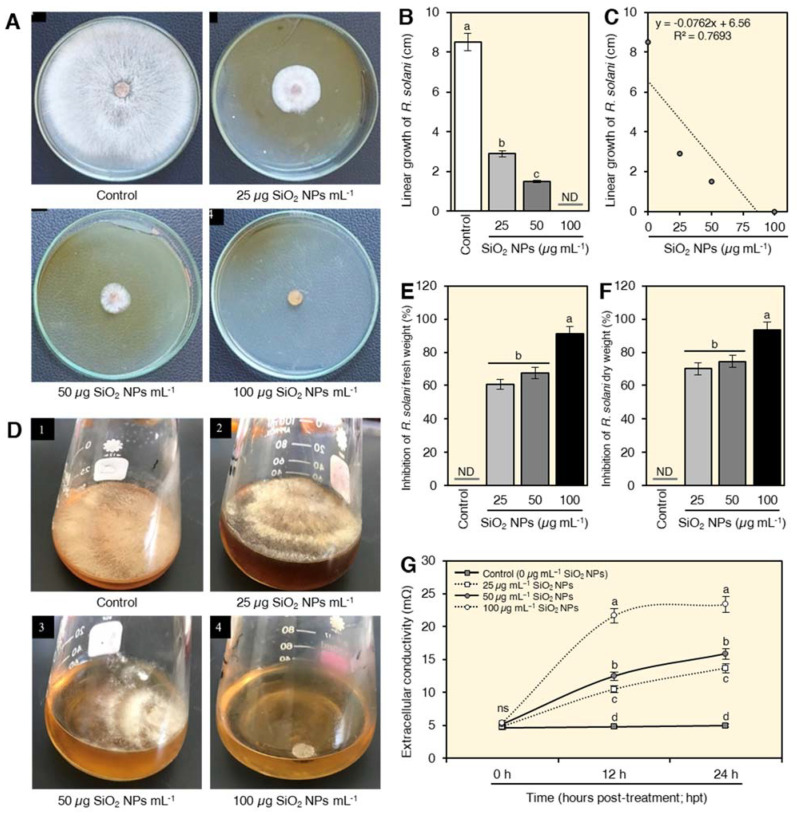
In vitro antifungal activity of silicon dioxide nanoparticles (SiO_2_ NPs) against R. solani, the causal agent of wheat damping-off. (**A**) Antifungal activity of different concentrations (0 [control], 25, 50, and 100 µg mL^−1^) of SiO_2_ NPs against R. solani growing on PDA medium in Petri dishes. (**B**) Linear growth (cm) of *R. solani* after the treatment with different concentrations of SiO_2_ NPs. (**C**) Simple linear regression between linear growth (cm) of *R. solani* and different concentrations of SiO_2_ NPs (0, 25, 50, and 100 µg mL^−1^). (**D**) Antifungal activity of different concentrations (0 [control], 25, 50, and 100 µg mL^−1^) of SiO_2_ NPs against R. solani growing on liquid medium. (**E**,**F**) Inhibition (%) of *R. solani* fresh and dry weight, respectively, after the treatment with different concentrations of SiO_2_ NPs. (**G**) Effect of different concentrations of SiO_2_ NPs on the extracellular conductivity of *R. solani* suspension at 0, 12, and 24 h post-treatment (hpt). The experiments were repeated twice independently. In bar graphs, data presented are means ± standard deviation (mean ± SD) of five biological replicates. Different letters indicate statistically significant differences among treatments, while the same letters signify no significant differences between them according to Tukey’s honestly significant difference test (*p* < 0.05). In simple linear regression graphs, the linear fit regression line is presented as a dotted line, regression equation and *R*^2^ are also presented within the graph.

The in vitro antifungal activity of biosynthesized SiO_2_ NPs was tested using two different methods (Figure 3). SiO_2_ NPs showed a strong dose-dependent fungistatic activity against *R. solani* since the higher concentrations showed a wider inhibition zone and vice versa (Figure 3A). The highest concentration of SiO_2_ (100 µg mL^−1^) showed no mycelial growth (100% growth reduction) of *R. solani* (Figure 3A,B). Applying SiO_2_ NPs at the three concentrations (25, 50 and 100 µg mL^−1^) significantly reduced the linear growth of *R. solani* (y = −0.0762x + 6.56, R^2^ = 0.7693; Figure 3C). Consequently, the inhibition (%) of the mycelial growth of *R. solani* was dose-dependent. No fungal growth was observed when mycelium was treated with 100 µg mL^−1^ SiO_2_ (100% growth reduction). Similarly, SiO_2_ NPs showed strong in vitro antifungal activity against *R. solani* growing on liquid media (Figure 3D) as expressed by inhibition of both mycelium fresh (Figure 3E) and dry weight (Figure 3F) of treated cultures. The highest concentration of SiO_2_ NPs (100 µg mL^−1^) was the most effective concentration in reducing the fresh and dry weight of *R. solani* mycelial growth by 91.0 and 93.5, respectively (Figure 3E and Figure 3F, respectively).

### 2.4. SiO_2_ NPs Increased the Extracellular Conductivity of Treated Fungal Suspension

Treating the mycelium of *R. solani* with SiO_2_ NPs significantly increased levels of extracellular conductivity compared to control. The conductivity was increased progressively by the time of exposure with a greater effect with the highest concentration of SiO_2_ NPs (100 µg mL^−1^). However, there was no significant effect between 12 and 24 h exposure time to the higher concentration of SiO_2_ on the conductivity of the supernatant (Figure 3G). 

### 2.5. SiO_2_ NPs Enhanced the Germination, Root, and Shoot Length, and Vigor Indexes of Wheat Plants in Soil Inoculation with R. solani

**Figure 4:**
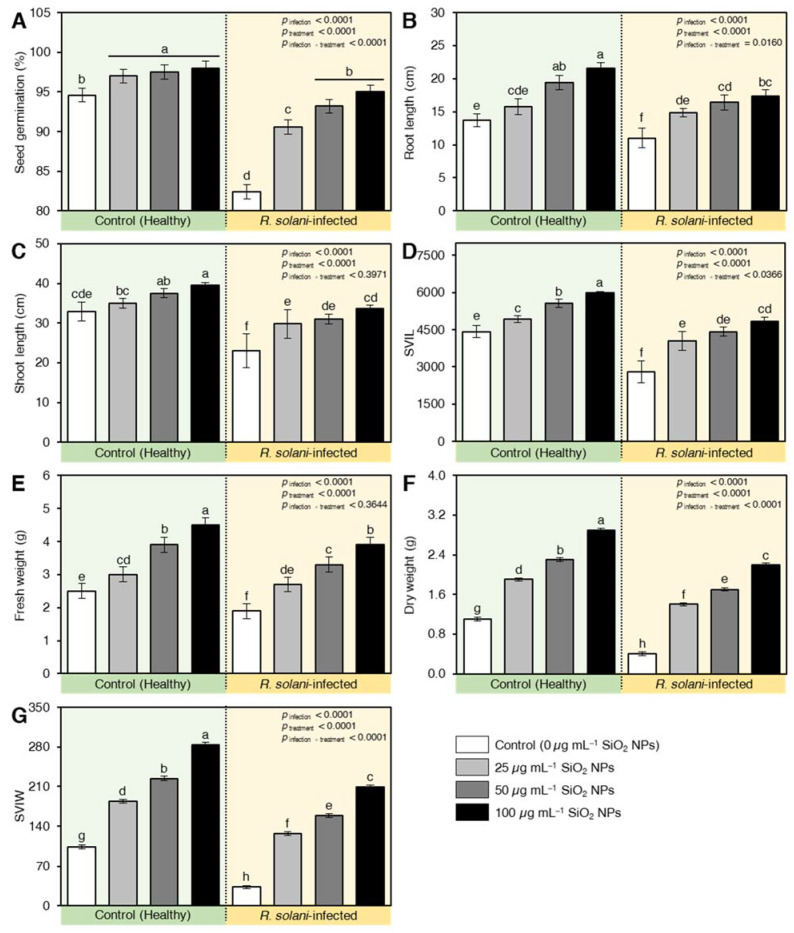
Effect of silicon dioxide nanoparticles (SiO_2_ NPs) on vegetative traits of wheat plants under soil inoculation with *R. solani*. (**A**) seed germination (%), (**B**) Root length, (**C**) Shoot length, (**D**) Seedling vigor index based on plant length (SVIL), (**E**) Fresh weight, (**F**) Dry weight, (**G**) Seedling vigor index based on plant wright (SVIW) of wheat seedlings infected with *R. solani*. Data presented are means ± standard deviation (mean ± SD) of five biological replicates. Different letters indicate statistically significant differences among treatments, while the same letters signify no significant differences between them according to Tukey’s honestly significant difference test (*p* < 0.05).

Treating wheat grains with SiO_2_ significantly increased the germinated grains compared with control (Figure 4A). Infected and treated grains with SiO_2_ NPs at 50 and 100 µg mL^−1^ showed a higher germination percentage compared with infected and non-treated seeds (positive control). The highest germination percentage 98% was observed in non-infected wheat seeds that were treated with SiO_2_ NPs at a concentration of 100 µg mL^−1^. No significant effect was found between the germination (%) of the negative control (untreated healthy seeds) and inoculated seeds that were treated with 100 µg mL^−1^ SiO_2_ NPs (Figure 4A). 

Likewise, SiO_2_ NPs application significantly enhanced both root (Figure 4B) and shoot lengths (Figure 4C) of both healthy and *R. solani*-infected seedlings. As a result, all tested concentrations of SiO_2_ NPs significantly increased the vigor index based on root and shoot length (SVIL; Figure 4D) in both healthy and infected wheat seedlings. Applying SiO_2_ NPs at 50 and 100 µg mL^−1^ on healthy wheat seeds significantly increased SVIL values to 5552.9 and 5983.7, respectively. Additionally, treating infected wheat seeds with 50 and 100 µg mL^−1^ SiO_2_ NPs resulted in SVIL values of 4419.6 and 4840.9, respectively, compared with 4413.4 for healthy untreated seedlings (negative control).

Similar results were observed in both seedling fresh weight (Figure 4E), seedling dry weight (Figure 4F), and when the seedling vigor index was measured based on seedling weight (SVIW; Figure 4G). The highest SVIW was noticed when healthy seeds were treated with 100 µg mL^−1^ SiO_2_ NPs (284.4) followed by 50 µg mL^−1^ (224.4). All tested concentrations of SiO_2_ NPs significantly increased SVIW of inoculated seedlings compared with negative and positive controls (Figure 4G).

### 2.6. SiO_2_ NPs Application Reduced the Disease Parameters of R. solani—Inoculated Wheat 

**Figure 5:**
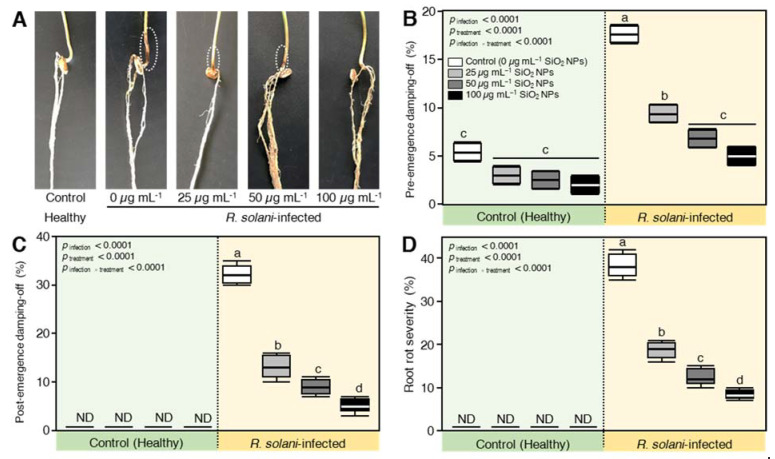
Effect of silicon dioxide nanoparticles (SiO_2_ NPs) on damping-off severity of wheat seedlings infected by *R. solani.* (**A**) Healthy (negative control), *R. solani*-infected (positive control), and SiO_2_-treated wheat seedlings, (**B**) pre-emergence damping-off, (**C**) Post-emergence damping-off, and (**D**) Root rot severity. Whiskers show the minimum and the maximum values, horizontal thick lines indicate the medians, boxes signify the interquartile ranges (25th to 75th percentile of the data). Different letters indicate statistically significant differences among treatments, while the same letters signify no significant differences between them according to Tukey’s honestly significant difference test (*p* < 0.05; *n* = 5).

Soil infestation with *R. solani* significantly elevated pre-, post-emergence damping-off, and root rot percentages on inoculated wheat seedlings. SiO_2_ NPs application showed reduced disease frequency and completely protected wheat seedlings at 100 µg mL^−1^ (Figure 5A). Untreated wheat seedlings grown in *R. solani-*infested soil showed 17.6% pre-emergence compared to 5.4% in untreated healthy seedlings (Figure 5B). *R. solani* caused a significant increase in post-emergence damping-off and root rot (Figure 5C,D) recording 32.2% and 38.4%, respectively, compared to 0% in the uninoculated negative control. In addition, treating the seeds with SiO_2_ NPs significantly decreased the percentages of pre, post-emergence damping-off, and root rot compared to inoculated positive control. The highest concentration of SiO_2_ NPs 100 µg mL^−1^ gave the lowest pre-emergence damping-off (5%), post-emergence damping-off (5.2%), and root rot (8.4%), followed by SiO_2_ NPs at 50 and 25 µg mL^−1^.

### 2.7. Effect of SiO_2_ NPs on the Biochemical Traits of Wheat Seedlings Inoculated with R. solani

#### 2.7.1. SiO_2_ NPs Enhanced the Content of the Photosynthetic Pigment in *R. solani*-Infected Plants

**Figure 6:**
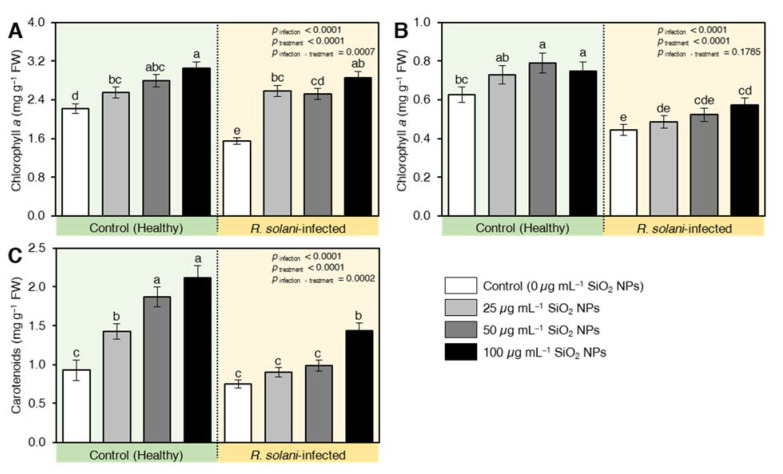
Effect of silicon dioxide nanoparticles (SiO_2_ NPs) on the content of photosynthetic pigments of wheat seedlings infected by *R. solani.* (**A**) Chlorophyll *a* (mg g^−1^ FW), (**B**) Chlorophyll *b* (mg g^−1^ FW), and (**C**) Carotenoids (mg g^−1^ FW). Data presented are means ± standard deviation (mean ± SD) of five biological replicates. Different letters indicate statistically significant differences among treatments, while the same letters signify no significant differences between them according to Tukey’s honestly significant difference test (*p* < 0.05).

Infection with *R. solani* adversely reduced the pigment content of infected wheat seedlings. In this regard, the chlorophyll *a* (Figure 6A), chlorophyll *b* (Figure 6B), and carotenoids (Figure 6C) were reduced in the positive control (infected untreated seedlings) by 30, 30, and 20% relative to the negative control (untreated uninfected seedlings). On the other hand, the seedlings treated with SiO_2_ NPS had a protective effect on wheat plants by alleviating the reduction of pigments contents higher than the healthy plants, which were witnessed at the level of 100 µg mL^−1^. The positive effect of SiO_2_ NPs was also reported on the healthy plants and the effect intensified as the concentration of SiO_2_ NPs increased (Figure 6). 

#### 2.7.2. Treatment with SiO_2_ NPs Alleviated the Oxidative Stress in *R. solani*-Infected Plants

**Figure 7:**
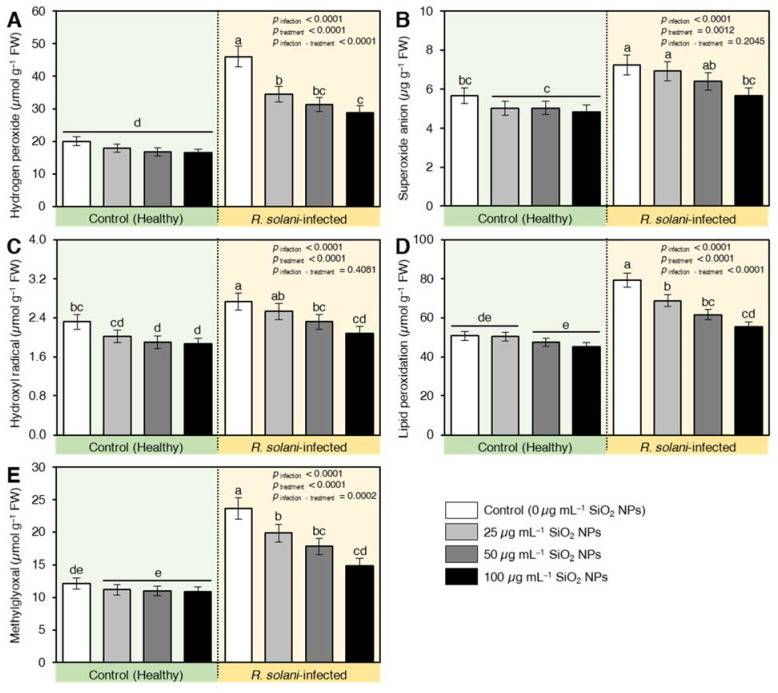
Effect of silicon dioxide nanoparticles (SiO_2_ NPs) on oxidative stress-related compounds of wheat seedlings infected by *R. solani.* (**A**) Hydrogen peroxide (µmol g^−1^ FW), (**B**) Superoxide anion (µg g^−1^ FW), (**C**) Hydroxyl radical (µmol g^−1^ FW), (**D**) Lipid peroxidation (µmol g^−1^ FW), and (**E**) Methylglyoxal (µmol g^−1^ FW). Data presented are means ± standard deviation (mean ± SD) of five biological replicates. Different letters indicate statistically significant differences among treatments, while the same letters signify no significant differences between them according to Tukey’s honestly significant difference test (*p* < 0.05).

Infection with *R. solani* induced a high accumulation of hydrogen peroxide (129%; Figure 7A), superoxide anions (27%; Figure 7B), and hydroxyl radicals (17%; Figure 7C) compared with healthy plants (negative control). This effect was retarded by the applied levels of SiO_2_ NPS where the values of reactive oxygen species were lower than healthy plants (Figure 7). Likewise, the results deduced that *R. solani* enhanced lipid peroxidation (56%; Figure 7D) and the contents of methylglyoxal (95%; Figure 7E) relative to healthy plants (Figure 7). It is worthy to mention that applying SiO_2_ NPs with different concentrations decreased the peroxidation of lipids and methylglyoxal compared to pathogen-infected plants and the lowest reduction was recorded for the level of 100 µg mL^−1^ where lipid peroxidation and methylglyoxal increased only by 7.3 and 18.8% higher than healthy plants and decreased by 30% and 36.8% compared with the infected control, respectively (Figure 7).

#### 2.7.3. SiO_2_ NPs Induced the Accumulation of Defense-Related Compounds in *R. solani*-Infected Seedlings

**Figure 8:**
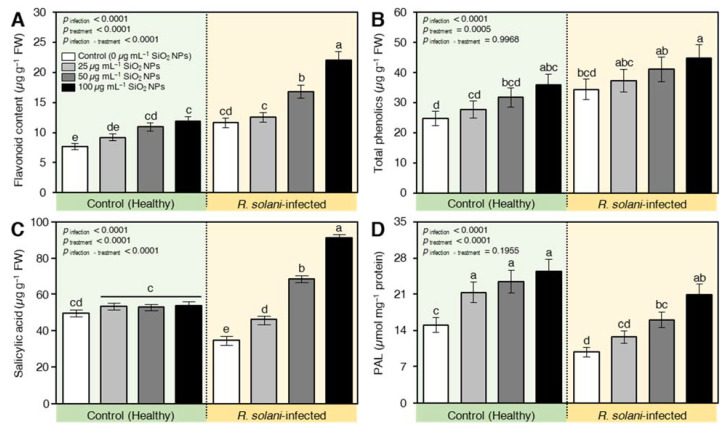
Effect of silicon dioxide nanoparticles (SiO_2_ NPs) on defense-related compounds in wheat seedlings infected by *R. solani.* (**A**) Flavonoid content (µg g^−1^ FW), (**B**) Total phenolics (µg g^−1^ FW), (**C**) Salicylic acid (µg g^−1^ FW), and (**D**) phenylalanine ammonia-lyase (PAL; a key SA biosynthesis enzyme) (µmol mg^−1^ protein). Data presented are means ± standard deviation (mean ± SD) of five biological replicates. Different letters indicate statistically significant differences among treatments, while the same letters signify no significant differences between them according to Tukey’s honestly significant difference test (*p* < 0.05).

It is worth mentioning that the infection with *R. solani* significantly increased secondary metabolites and defense-related compounds. The infection increased the contents of total flavonoids (Figure 8A) and total phenolics (Figure 8B) by about 54 and 38%, respectively, relative to the healthy plants. However, the utilization of different levels of SiO_2_ NPs instigated the secondary metabolites in healthy and infected plants. The exposure of seedlings to 100 µg mL^−1^ had a prominent role relative to other levels for inducing secondary metabolites (Figure 8). At the same time, the application of SiO_2_ NPs significantly enhanced the accumulation of salicylic acid in infected seedlings, but not healthy ones (Figure 8C). The application of SiO_2_ NPs at the levels of 50 and 100 µg mL^−1^ elevated the reduction in SA content in infected plants to a higher level than in healthy plants. Moreover, the activity of phenylalanine ammonia-lyase (PAL; a key SA biosynthesis enzyme) was negatively affected by *R. solani*. The applied protectant increased the PAL activity, and the enhanced value was intensified as the concentration of SiO_2_ NPs increased (Figure 8D).

#### 2.7.4. SiO_2_ NPs Stimulated the Non-Enzymatic Antioxidant Machinery of *R. solani*-Infected Seedlings

**Figure 9:**
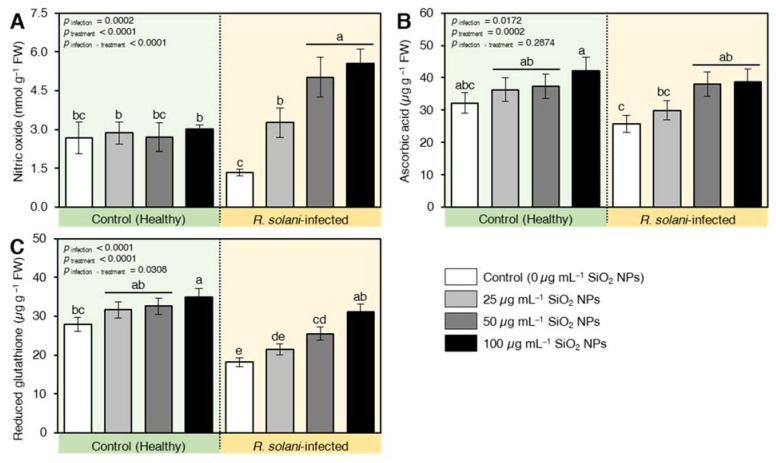
Effect of silicon dioxide nanoparticles (SiO_2_ NPs) on the non-enzymatic antioxidant machinery of *R. solani*-infected seedlings. (**A**) Nitric oxide (nmol g^−1^ FW), (**B**) Ascorbic acid (µg g ^−1^ FW), and (**C**) Reduced glutathione (µg g ^−1^ FW). Data presented are means ± standard deviation (mean ± SD) of five biological replicates. Different letters indicate statistically significant differences among treatments, while the same letters signify no significant differences between them according to Tukey’s honestly significant difference test (*p* < 0.05).

The non-enzymatic antioxidants, such as nitric oxide (Figure 9A), ascorbic acid (vitamin C; Figure 9B), and glutathione (GSH; Figure 9C) were found at lower levels in nontreated *R. solani*-infected seedlings. However, these harmful effects were neutralized upon SiO_2_ NPs application (Figure 9) in infected plants. For instance, nitric oxide (NO) recorded lower values for only *R. solani*-infected plants. The used applicants increased the NO content of wheat leaves compared to infected plants only (Figure 9A). The highest induction was recorded also for the level of 100 µg mL^−1^ SiO_2_ NPs. Likewise, the pool of ascorbic acid content was significantly increased by the application of SiO NPs. Intriguingly, ascorbic acid recorded a high value under the interactive effect of 100 µg mL^−1^ SiO_2_ NPs and pathogen-infected plants compared with infected and nontreated control (positive control) (Figure 9B). Similarly, reduced glutathione (GSH) in the infected plants seems to be deregulated by *R. solani* infection where the lowest values were obtained. The GSH levels were increased after the application of nanomaterial with high alleviation capacity at the concentration of 100 µg mL^−1^ SiO_2_ NPs (Figure 9C). 

#### 2.7.5. SiO_2_ NPs Induced the Enzymatic Antioxidant Machinery of *R. solani*-Infected Seedlings

**Figure 10:**
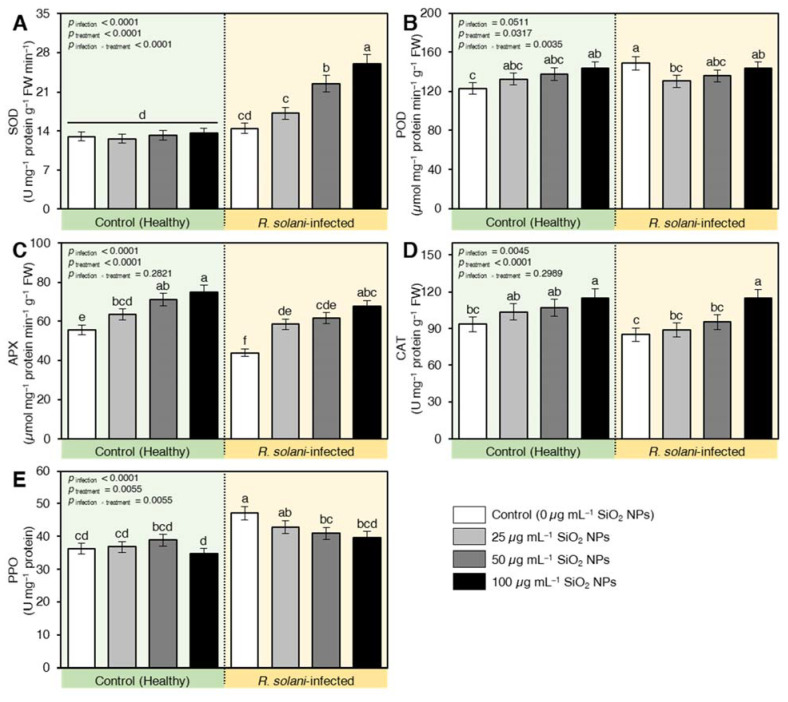
Effect of silicon dioxide nanoparticles (SiO_2_ NPs) on enzymatic antioxidant machinery of *R. solani*-infected seedlings. (**A**) Superoxide dismutase (SOD), (**B**) Guaiacol peroxidase (POD), (**C**) Ascorbate peroxidase (APX), (**D**) Catalase (CAT), and (**E**) Polyphenol oxidase (PPO). Data presented are means ± standard deviation (mean ± SD) of five biological replicates. Different letters indicate statistically significant differences among treatments, while the same letters signify no significant differences between them according to Tukey’s honestly significant difference test (*p* < 0.05).

The antioxidant enzymes responded differentially to *R. solani* infection. SOD (Figure 10A) and POD (Figure 10B) activities were increased by 11 and 46%, respectively, relative to healthy plants. On the other hand, the activities of both APX (Figure 10C) and CAT (Figure 10D) were reduced by 21 and 10%, respectively, due to *R. solani* infestation. Nevertheless, the application of SiO_2_ NPs induced the antioxidant enzymes by restricting the reduction of APX and CAT and exacerbated the content of SOD compared to infected and healthy plants. It is worth mentioning that the activity of POD was kept at the level of infected plants under the interactive effect of SiO_2_ NPs and *R. solani* infection, but higher than control plants. The healthy plants treated with SiO_2_ NPs recorded higher activities of POD, APX and CAT compared to non-treated healthy plants. The activity of SOD in infected plants was kept at levels similar to the control irrespective of the level of SiO_2_ NPs applied (Figure 10). Similarly, polyphenol oxidase (PPO) activity was found to be at the lowest content for healthy plants receiving no nano-silicon treatments (Figure 10E). On the other hand, *R. solani* enhanced the PPO activity and the application of SiO_2_ NPs reduced PPO in infected plants compared with infected and nontreated control especially at the highest concentrations 100 µg mL^−1^.

## 3. Discussion

Spherical shaped nano silicon dioxide (SiO_2_) particles were successfully biosynthesized during this study using saffron extraction. The role of saffron in the biosynthesis of metal oxides nanoparticles was explained as the extract of saffron petals contains hydroxyl groups from several phenolic compounds, such as flavonoids like kaempferol and anthocyanins like anthocyanidin, delphinidin, and pelargonidin. These hydroxyl groups enhance saffron’s ability for the biosynthesis of metal NPs, as it binds with metal ions and acts as a reducing agent for the reduction of metal crude particles to metal NPs [31,32]. It was previously inferred that biosynthesis of nanoparticles improves the physicochemical characteristics of particles, such as the surface area-to-volume ratio and enhances the stability of the structure that improves the anti-microbial activity of the nanometals against plant pathogens. 

In this study, SiO_2_ NPs were biosynthesized using saffron extract. The maximum peak of SiO_2_ NPs was observed at approximately 440 nm, which is almost the same finding as reported by Tripathi et al. [33]. FTIR spectroscopy uses wavelength to measure the absorption of infrared radiation by tested samples, the reading of the infrared spectrum includes the interpretation of the correlation between the absorption bands (vibration bands) and the chemical compounds in the sample. The spectra of SiO_2_ NPs showed a band around 1103 cm^−1^ which corresponds to the asymmetric stretching vibration of Si-O-Si [34,35,36]. A wide absorption range was observed at 1634 and 3405 cm^−1^ that indicates the presence of H-O-H stretching and bending of absorbed water [37,38]. The observed peaks at 801 cm^−1^ are assigned to the Si-OH bond [34,36].

The broadband detected with XRD which showed reflection at 2Ѳ = 22.8^o^ and the strong absorption band at 440 nm by UV-vis indicated that amorphous SiO_2_ nanoparticles were successfully synthesized [33,39]. Additionally, no additional peaks were observed in the XRD spectrum which indicates there are no impurities in the obtained SiO_2_ NPs. Additionally, the percentage of each element was calculated using the energy dispersion spectrum (EDS) which showed that SiO_2_ NPs contained 29% Si and 71% O. SiO_2_ NPs sized between 20 and 95 nm were synthesized by sol-gel method using sodium silicate [33]. According to UV-Vis, FTIR, XRD, and TEM images, using saffron extraction in the biosynthesis of SiO_2_ NPs resulted in smaller particle sizes 9.92 to 19.8 nm with safer and fewer coast methods. 

The importance of SiO_2_ NPs lays in their dual effect on both pathogen and host plant. On the pathogen side, silicon dioxide nanoparticles showed high antifungal activity against *R. solani* and completely inhibited its growth at 100 µg mL^−^^1^. The antifungal effect of SiO_2_ NPs against the human pathogenic *Trichoderma Harzianum* and *Rhizoctonia* species was previously reported by Verma and Bhattacharya [35]. Moreover, Derbalah et al. stated that SiO_2_ NPs showed an antifungal effect against Alternaria solani, which significantly inhibited the growth of Alternaria mycelium and reduced the severity of early blight disease on tomato plants [40]. 

Various theories explained the fungicidal effect of silicon nanoparticles. The accumulation of SiO_2_ NPs in the membrane of the fungal cell wall may result in the lysis of the cells, blockage of the transmembrane energy cycle, disruption of the electron transport chain, or oxidation of the cell membrane by forming insoluble compounds in the cell membrane. SiO_2_ NPs interact with protein thiol groups (-SH) on the fungal cell surface leading to cell lysis due to its positive charge [40]. Silicon nanoparticles may deactivate the protein molecules and directly bind with fungal DNA causing mutation and affecting replication [41]. Furthermore, hydroxyl groups on the surface of small particles of silicon bind with lipopolysaccharides of the fungal cell wall and cause a breakdown of treated fungal cells [42]. In this study, the loss of integrity of *R. solani* cell wall and cell membrane was confirmed by leakage of cellular materials, which resulted in changes in extracellular conductivity.

On the host plant side, It is well-documented that silicon application positively increases the host plant resistance against phytopathogens and plays important role in the interaction between the host plant and the pathogen [43]. Additionally, it decreases the severity of fungal diseases and induced resistance against a wide range of plant pathogenic fungi that cause root and foliar diseases [25,44]. It is worth mentioning that biosynthesized SiO_2_ NPs showed decreased pre-, post-emergence damping-off and root rot percentages of infected wheat seedlings in the current study. This might be due to the negative effects of SiO_2_ NPs on the fungal pathogen since our findings showed a strong dose-dependent fungistatic activity against *R. solani in vitro.* For instance, the highest concentration of SiO_2_ (100 µg mL^−1^) showed no mycelial growth (100% growth reduction) of *R. solani*. 

Moreover, the suppression of pre-, post-emergence damping-off and root rot severity caused by SiO_2_ NPs could be attributed to its contribution to different defense mechanisms. One of the defense mechanisms that could be expressed by silicon is the formation of physical barriers through Si accumulation below the cuticle and in cell walls, which contributed to preventing or delaying the penetration of infection pegs of *R. solani* appressoria [45,46]. Additionally, silicon effectively prevents fungal ingress by the formation of a Si-enriched layer and the uniform distribution of Si aggregates [43,44]. 

The accurate biological mechanisms of potential interaction between silicon and different biochemical pathways that lead to plant resistance to fungal pathogens remain unclear [46]. The defense mechanisms induced by silicon involve rapid production of defense compounds, i.e., phenolics, flavonoids, anthocyanins, lignin, callose, and phytoalexins through primary, as well as secondary metabolic pathways, also silicon increased activities of defense enzymes, such as phenylalanine ammonia-lyase (PAL), polyphenol oxidase (PPO), peroxidase (POX), lipoxygenase (LOX), chalcone synthase (CHS) chalcone isomerase, β-1,3-glucanase (GLU), pathogenesis-related (PR) proteins and chitinases [47,48,49]. Interestingly, our findings showed that SiO_2_ NPs application simultaneously activated multiple defense mechanisms in treated wheat seedlings. These mechanisms involve (i) the induction of defense-related compounds, particularly SA accumulation and its major biosynthetic enzyme PAL, and (ii) activation of both enzymatic (POD, SOD, APX, CAT, and PPO) and non-enzymatic (phenolics and flavonoids) antioxidant defense machinery.

In addition, the alteration of membrane phospholipids acts as signaling components during *R. solani* infection. In the current study, the infected plants suffered from high damage of the membrane by exacerbation of lipid peroxidation content, which was repeatedly outlined in the literature as an indicator of the oxidative burst in stressed plants [50]. Methylglyoxal (MG) accumulation was shown to be correlated with increased intracellular oxidative stress, due to enhanced reactive oxygen species (ROS) production [50]. The studied plants encountered a high accumulation of MG content under *R. solani* infection. 

The excessive MG accumulation in plant cells under infection by *R. solani* can inhibit cell proliferation, chlorophyll biosynthesis, instigate the deactivation and/or degradation of proteins, as well as antioxidant defending products, leading to disruption of many cellular functions [51]. All these deteriorations induced by *R. solani* were augmented by SiO_2_ NPS. The SiO_2_ NPs upregulated the content of MG and lipid peroxidation concomitant with reduction of ROS and augmentation of antioxidants and chlorophyll content. Thus, SiO_2_ NPs triggered better intact membrane structure through the reduction of the fluid structure of membrane lipids. The situation was confirmed by lower ROS, lipid peroxidation, and methylglyoxal activity. Thus, wheat plants respond to nanoparticles by fine-tuning redox homeostasis and so resist *R. solani* infection. Si and Si nanoparticles might play an important role in promoting protective mechanisms to decline membrane lipid peroxidation and electrolyte leakage through NO-signaling [33].

Moreover, the interaction between salicylic acid (SA) and reactive oxygen species (ROS) is well-documented [52]. The SA-ROS plays a key role in the transcriptional reprogramming that occurs during the plant defense responses. Briefly, biotic stress, such as exposure to phytopathogenic fungi, triggers ROS production mainly at the apoplast [52]. As a result, plants try to defend themselves via multilayered defense mechanisms in a spatio-temporal manner via an SA-mediated pathway [52]. It is worth mentioning that SA not only has a pro-oxidant role, but it also plays an antioxidant role concertedly with glutathione (GSH) [52]. Interestingly, our findings showed that while infection with *R. solani* induced a high accumulation of hydrogen peroxide, superoxide anions, and hydroxyl radical compared with healthy plants, SiO_2_ NPs application reduced the ROS levels and induced the accumulation of defense-related compounds, particularly SA, in *R. solani*-infected seedlings. Collectively, these findings suggest that SiO_2_ NPs application might activate a feed-forward loop between ROS and SA in response to pathogen infection. However, further studies are required to better understand the molecular mechanisms of SiO_2_ NPs in the stimulation of SA-ROS interplay. 

Furthermore, the utilization of SiO_2_ NPs had a regulatory role in stimulating the biosynthesis of ascorbate as an antioxidant molecule that keeps ROS (superoxide anion, hydrogen peroxide, and hydroxyl radical) at lower levels than the infected plants in a concentration-dependent manner where 100 mg L^−1^ was the most effective concentration. Similarly, our findings showed that *R. solani* infection shifted the cellular redox balance toward a more oxidative state, including a decline of the GSH pool, affecting all cellular compartments, and ROS detoxification. A similar reduction was reported for onion under *Fusarium oxysporum* infection [53]. 

Moreover, our findings showed that although *R. solani* significantly stimulated the content of the superoxide anions, the activity of SOD was enhanced upon SiO_2_ NPs application. On the other hand, the high accumulation of H_2_O_2_ was ascribed to the reduction of CAT and APX activities in non-treated infected plants, suggesting the sensitivity of the studied plants to infection by *R. solani* and breakdown of main defense strategies, which weaken the resistance of plants. However, SiO_2_ NPs application significantly induced the activities of SOD, CAT, and APX in connection with the reduction of disease severity, ROS, and membrane deterioration traits (lipid peroxidation and methyloxyal). Furthermore, enhanced antioxidant enzymes activities played a key regulatory role in plant growth, photosynthetic rate, cellular redox potential, and membrane integrity by eliminating free radicals [54]. Our findings showed that Si NPs significantly improved activities of SOD, APX, CAT, and GPX, which further counterbalance ROS as indicated by lower lipid peroxidation and electrolyte leakage [33]. Collectively, our findings indicate that SiO_2_ NPs enhance the wheat defense response against *R. solani* via upregulation of both enzymatic and non-enzymatic antioxidant defense machinery.

Apart from their role in catalyzing the breakdown of H_2_O_2_, PODs were reported to be involved in lignification and suberization processes [55]. POD activity of all SiO_2_ NPs-treated plants under stress was lower than that of the control levels. Decreased activity of POD revealed that using SiO_2_ NPs might reduce lignification processes resulting from *R. solani* infection. Therefore, SiO_2_ NPs can reduce stress severity where there is no need for high lignification as reported by Bagy et al. [55] who reported that mycorrhizal fungi and entophyte reduce the content of soluble and ionic peroxidases and lignification under *Pectobacterium* infection. 

In the present study, *R. solani* stimulated the production of phenolic compounds of wheat plants compared to healthy plants concomitant with enhanced peroxidase activity. Thus, the accumulation of phenolic compounds is a positive sign of stimulation of resistance by SiO_2_ NPs, which significantly exacerbated phenolic compound production higher than in infected plants. Using nano-silica increased the contents of phenolics similar to the studies of [56,57]. Moreover, SiO_2_ NPs application significantly induced the accumulation of defense-related compounds in *R. solani*-infected seedlings, particularly SA. SA is a defense-associated phytohormone that is mainly involved in plant responses to biotrophic and necrotrophic pathogens [58,59,60,61]. Furthermore, the major SA-biosynthetic enzyme, PAL, was dramatically reduced by infection; however, SiO_2_ NPs promptly augmented the reduction of PAL under stress and further enhanced the activity of PAL, which is concomitant with the high induction of phenolic compounds and flavonoids. PAL is involved in the synthesis of plant secondary antimicrobial substances and it is essential for plant disease resistance responses [53] and plays an essential role in the biosynthesis of the precursors of lignin [62].

Plant defense via lignification is a conserved basal mechanism in the plant immune response against pathogens [63]. Silicon increases the transcript abundance of PAL in rice plants, leading to enhanced lignification [64], while SiO_2_ NPs enhance PAL expression and lignification in the leaves and roots of oat seedlings [65]. The reduction of disease severity in rice [66] and cucumber [67] by silicon pre-treatment has been attributed to an increase in the activities of PPO and PAL. Therefore, the increase in the activity of PPO and PAL in the present study could prevent the pathogenic infection in beetroot due to silicon supplementation. 

PPO activity was significantly stimulated upon treatment with SiO_2_ NPs during fungal infection compared with healthy plants. This role of SiO_2_ NPs revealed that the SiO_2_ NPs reduced the pathogenesis, so the need for higher PPO in infected plants is not needed. PPO is a defense enzyme associated with fostering host resistance because it can catalyze the final step of lignin biosynthesis [62], and oxidize phenolic compounds to quinones [55]. The resulting quinones are toxic to invading organisms [68], hence possessing antipathogenic properties [69]. Consequently, SiO_2_ NPs treatment reduces the severity of invading microorganisms and enhances suitable defending agents that support plant immunity. POD, PPO, and PAL defense enzymes are generally working together for enhancing plant resistance to pathogens. This conclusion was recommended for infected plants treated with SiO_2_ NPs that had adequate PPO and PAL activities, which aided in preventing further development of infection.

## 4. Materials and Methods

### 4.1. Biosynthesis of Silicon Dioxide (SiO_2_) Nanoparticles

#### 4.1.1. Preparation of Aqueous Saffron Extraction

The aqueous saffron extraction was prepared as described by Nagaonkar et al. [70] with slight modification. Briefly, one gram of dried saffron stigmas (Qingdao Jingtai Huacheng International Trad Co., Ltd., Qingdao, Shandong, China) was suspended in 100 mL of deionized water. The mixture was homogenized and kept at 60 °C for 6 h. The obtained extract was filtered three times using Whatman filter paper no. 1 and further used in the process of synthesis of SiO_2_ NPs.

#### 4.1.2. Synthesis of SiO_2_ Nanoparticles

SiO_2_ NPs were prepared following the protocols described previously [71,72,73] with slight modifications. Briefly, 100 mL of the aqueous saffron extract was mixed with 100 mL of 0.1 M bulk SiO_2_ (Qingdao Jingtai Huacheng International Trad Co., Ltd. Model No. XR.16, Silicon Dioxide) solution. The prepared solution was stirred at 60 °C for 6 h, then centrifuged at 10,000 rpm for 10 min. The supernatant was discarded and the pellets were collected and lyophilized to obtain the powder.

#### 4.1.3. Characterization of SiO_2_ Nanoparticles

##### Analysis of Ultraviolet Spectra 

SiO_2_ NPs were checked at a wavelength ranging from 200 to 800 nm for the highest peak absorption using UV-Vis spectroscopy (Shimadzu Spectrometer, Shimadzu Corporation, Kyoto, Japan) [74]. 

##### Fourier Transform Infrared Spectroscopy (FTIR)

Formation of SiO_2_ NPs was detected in the collected powder using FTIR analysis at a range of 400–4000 cm^−1^ regions using Fourier transform infrared spectrometer (Vector 22, Bruker, Germany) at a resolution of 4 cm^−1^.

##### X-ray Diffraction (XRD)

The purity of extracted particles was tested using an X-ray diffractometer (XRD) by fixing a coated film of dried SiO_2_ NPs powder on glass slides with operating conditions of 45 kV and 20 mA current with Cu-Ka radiation as an X-ray source in the 20–80° range at the 2Ѳ angle.

##### Transmission Electron Microscopy (TEM)

The transmission electron microscope JEM-1230, JEOL, Akishima, Japan, was used to detect the morphology of SiO_2_ nanoparticles. The samples were processed as described by Atallah et al. [75]. A film from the sample was placed in a grid box and the procedures recommended by the manufacturer were followed.

### 4.2. Pathogen Isolate and Pathogenicity Test

An isolate of *R. solani* was obtained from the Department of Plant Pathology, Minia University, Egypt. The isolate was previously identified at Molecular Biology Research Unit, Assiut University and SolGent Company, Daejeon South Kore based on sequence analysis of the internal transcribed spacer (ITS) region using ITS1 and ITS4 primers. The *R. solani* isolate was propagated on a PDA medium, incubated for seven days at room temperature (28 ± 2 °C), and used as an inoculum source for the inoculation of wheat seeds [76,77].

The pathogenicity of *R. solani* isolate was tested on eight wheat cultivars namely Gemmiza-11, Gemmiza-12, Giza-168, Giza-171, Misr-1, Misr-2, Sids-13, and Sids-14. Briefly, sterilized soil was added to three-inches diameter pots. The inoculum of *R. solani* was cultured on barley grains was mixed with the soil at 1% (W: W). The infested pots were watered as needed and left for five days to enhance the establishment of *R. solani* growth in the soil. 1% sodium hypochlorite solution was used to surface sterilize the seeds of each wheat cultivar by immersing the seeds for 2 min in the solution. The seeds were then rinsed in sterile distilled water. Three seeds were sown in each pot. Five replicates were used for each treatment. Pots filled with non-infested soil were used as a negative control. The pots were randomly distributed within each plot. Pre-, post-emergence damping-off and root rot for wheat seedlings were evaluated two, four, and six weeks after the sowing date, respectively, according to Raju and Naik [78] and Beale et al. [79].

The pre- and post-emergence damping-off was calculated using Equation (1) as follows:

(1) $$\mathrm{Damping}-\mathrm{off}\;\left(\%\right)=\frac{\mathrm{No}.\;\mathrm{of}\;\mathrm{infected}\;\mathrm{plants}}{\mathrm{total}\;\mathrm{No}.\;\mathrm{of}\;\mathrm{plants}}\;\times \;100$$

Furthermore, the disease severity of root rot was assessed using the indexing method described by Beale et al. [79]. The roots of wheat plants were washed and divided into five categories according to the percentage of all roots with lesions typical of root rot: zero (0) trace to 10% (1), >10% and ≤30% (2), >30% and ≤60% (3), and >60% (4). Disease severity percentage was calculated using Equation (2) as follows:

(2) $$\mathrm{Disease}\;\mathrm{severity}\;\left(\%\right)=\frac{(i\;(\mathrm{rating}\;\mathrm{no}.\;\times \;\mathrm{no}.\;\mathrm{of}\;\mathrm{plants}\;\mathrm{in}\;\mathrm{the}\;\mathrm{rating})}{(\mathrm{total}\;\mathrm{no}.\;\mathrm{of}\;\mathrm{plants}\;\times \;\mathrm{highest}\;\mathrm{rating})}\;\times \;100$$

### 4.3. In Vitro Antifungal Activity of SiO_2_

#### 4.3.1. Effect of SiO_2_ NPs on the Linear Growth of *R. solani*

The experiment was implemented as described by Joshi et al. [80] with minor modifications. Potato dextrose agar (PDA) medium was prepared, and SiO_2_ NPs were amended into the media to obtain a final concentration of 25, 50, and 100 µg mL^−1^. The media was poured into 9 cm diameter Petri dishes. After solidification, a 0.5 cm diameter disc of 5 days old *R. solani* culture was placed at the center of the dishes. The reduction percentage of pathogen growth was calculated using Equation (3) [81,82] as follows:

(3) $$\mathrm{Inhibition}\;\mathrm{of}\;\mathrm{pathogen}\;\mathrm{growth}\;(\%)=\frac{\mathrm{growth}\;\mathrm{in}\;\mathrm{the}\;\mathrm{control}\;-\;\mathrm{growth}\;\mathrm{in}\;\mathrm{the}\;\mathrm{treatment}}{\mathrm{growth}\;\mathrm{in}\;\mathrm{the}\;\mathrm{control}}\;\times \;100$$

#### 4.3.2. Effect of SiO_2_ NPs on the Mycelium Fresh and Dry Weight of *R. solani*

Mycelium dry weight was assessed according to the method described by Li et al. [83] with minor modifications. 50 mL of potato dextrose broth (PDB) medium was poured into a 250 mL Erlenmeyer flask. SiO_2_ NPs were added to the media and the concentration was adjusted to 25, 50, and 100 µg mL^−1^. No SiO_2_ NPs were added to the control; 0.5 cm diameter disc of 5 days old *R. solani* culture was placed gently on the surface of PDB media. The inoculated flasks were incubated at 28 ± 2 °C for 5 days. For fresh weight assessment, the mycelium of *R. solani* from liquid media was harvested using a filtration-based method as previously described by Newell and Statzell-Tallman [84] with slight modifications. Briefly, the fungal mycelium was filtrated using a 40-µm-mesh nylon screen, washed with 25 mL distilled water, recollected on Whatman filter paper no. 1, then vacuumed for 30 s to abandon any free water from the samples. Subsequently, mycelia were kept in small Petri dishes to avoid rapid dehydration during fresh-weighting [84]. Fresh weight was assessed immediately after filtration. Samples were processed one by one through filtration and fresh-weighting. For dry weight assessment, the mycelia of *R. solani* left to dry at 50 °C for 24 h and reweighted. During the dry weight assay, fresh desiccant was maintained in the weighting chamber of the balance. The percentage of mycelium fresh and dry growth reduction was calculated according to Equation (4) as follows:

(4) $$\mathrm{Inhibition}\;\mathrm{of}\;\mathrm{mycelium}\;\mathrm{fresh}/\mathrm{dry}\;\mathrm{weight}\;\left(\%\right)=\frac{\mathrm{Mycelium}\;\mathrm{fresh}/\mathrm{dry}\;\mathrm{weight}\;\mathrm{of}\;\mathrm{control}\;-\;\mathrm{Mycelium}\;\mathrm{fresh}/\mathrm{dry}\;\mathrm{weight}\;\mathrm{in}\;\mathrm{the}\;\mathrm{treatment}}{\mathrm{Mycelium}\;\mathrm{fresh}/\mathrm{dry}\;\mathrm{weight}\;\mathrm{of}\;\mathrm{control}}\;\times \;100$$

Five replicates were used for each concentration each replicate was represented with one flask. The experiment was repeated twice. 

#### 4.3.3. Extracellular Conductivity

Extracellular conductivity assay was carried out using a method described by Mahas and Kaur [85] with minor modifications. To obtain the fungal mycelium, 50 mL of nutrient broth (NB) was inoculated by a 6 mm mycelial disk of five days old *R. solani* culture. The inoculated NB was incubated for three days at 26 °C. Sterile distilled water was used to wash the collected mycelium. 100 mg of collected mycelium was transferred into 50 mL PDB containing three concentrations of 25, 50, and 100 µg mL^−1^ of SiO_2_ NPs, and no particles were added to the control. The supernatants were collected at 0, 12, and 24 h of incubation by centrifuging the mycelial suspensions for 10 min at 10,000 rpm. The electrical conductivity of the supernatants was measured using a conductivity meter.

### 4.4. Effect of SiO_2_ NPs on Germination, Root and Shoot Length, and Vigor Indexes of R. solani-Infected Wheat Seedlings

This experiment was conducted to study the effect of three SiO_2_ NPs concentrations, 25, 50, and 100 µg mL^−1^, on the germination percentage, root length, shoot length, and vigor index of wheat cv. Misr-2 infected with *R. solani*. Inoculation with fungal isolate was the main plot, and SiO_2_ NPs treatment was the subplot. Five replicates were used for each treatment; three wheat seeds were sown in each pot as one replicate. The seeds of wheat cultivar Misr-2 were soaked in three concentrations of SiO_2_ NPs, 25, 50, and 100 µg mL^−1^ for 12 hr. The seeds were then divided into two groups; the first group was sown in 15 pots, which were previously inoculated with *R. solani* as described in the cultivar evaluation test. Five pots were used for each concentration of SiO_2_ NPs. The second group was sown in the same way except the soil was not inoculated with *R. solani.* Two controls were used in this experiment; Negative control (wheat seeds were soaked in water and sown in non-inoculated soil), Positive control (wheat seeds were soaked in water and sown in inoculated soil).

Two weeks after sowing dates, the wheat seedling was taken out of the pots and the roots were gently washed. The percentage of germinated seeds, roots, and shoots length, fresh and dry weight of wheat seedlings were measured. The germination percentage was calculated using Equation (5):

(5) $$\mathrm{Germination}\;\left(\%\right)=\frac{\mathrm{Number}\;\mathrm{of}\;\mathrm{germinated}\;\mathrm{seeds}}{\mathrm{Number}\;\mathrm{of}\;\mathrm{total}\;\mathrm{seeds}}\;\times \;100$$

Moreover, the vigor index of wheat seedlings was calculated using two traits. Seedling vigor index based on the root and shoot length (SVIL) was measured according to Equation (6) as described by Abdul-Baki and Anderson [16] as follows:

(6) SVIL=(shoot length+root length) × germination (%)

Likewise, the seedling vigor index based on the weight was calculated using Equation (7) as follows:

(7) SVIW=seedling dry mass at the end of the test × germination (%)

### 4.5. Effect of SiO_2_ on Pre- and Post-Emergence Damping-Off and Root Rot of Wheat under Inoculation with R. solani

Wheat cultivar Misr-2 was used for this test. Wheat seeds were treated with SiO_2_ NPs at three concentrations, 25, 50, and 100 µg mL^−1^ for 12 h. The treatments and experiment design were implemented as described in the above section. The inoculation was done as described in the cultivar evaluation test section. Pre-, post-emergence damping-off and root rot for wheat seedlings were evaluated two, four, and six weeks after the sowing date, respectively, as described previously.

### 4.6. Stress-Associated Biochemical and Physiological Assessments

The experiment was conducted and designed in the same way as described in the vigor index section. Leaves of three weeks old wheat seedlings from each treatment were used for the following measurements and five biological replicates were used for each trait.

#### 4.6.1. Pigments Content

Chlorophyll a, b and carotenoids were measured in fresh leaves (0.05 g) suspended in 5 mL of ethyl alcohol (95%) using equations recommended by Lichtenthaler [86].

#### 4.6.2. Reactive Oxygen Species

Reactive oxygen species (ROS) were assessed by determination of superoxide anions O_2_^─^^●^ (µg g^−1^ FW), hydrogen peroxide (µmol g^−1^ FW, H_2_O_2_), and hydroxyl radicals (^●^OH, µmol g^−1^ FW) via published methods of Yang et al. [87], Mukherjee and Choudhuri [88], and Halliwell et al. [89], respectively.

#### 4.6.3. Membrane Damage Criteria

Lipid peroxidation was detected as described by Madhava Rao and Sresty [90] with some modifications. The thiobarbituric acid reaction was used to determine lipid peroxidation in wheat shoots by monitoring malondialdehyde formation as explained Methylglyoxal (MG) was estimated based on the method of Gilbert and Brandt [91].

#### 4.6.4. Resistance-Related Compounds and Enzymes

##### Salicylic Acid (SA)

Fresh wheat leaves were used for the determination of SA as described by Warrier et al. [92]. Briefly, 100 mg of leaf tissue were ground to a fine powder using liquid nitrogen, extracted with 1.0 mL of extraction solvent (chloroform, amyl alcohol, ether, and ethanol), then centrifuged at 10,000× *g* for 10 min. subsequently, 100 μL of the supernatant with up to 3 mL of freshly prepared ferric chloride (0.1%) till the development of violet color. The absorbance of the complex was measured using spectrophotometry at 540 nm [92].

##### Phenolic Compounds

Phenolic compounds were determined using a Folin–Ciocalteu-based method as described by Kofalvi and Nassuth [93], using gallic acid as a standard curve. Total phenolics were expressed as mg g^−1^ FW. Briefly, 300 mg fresh leaf tissues were extracted in methanol (50%) in a water bath (70 °C) for one hour. The methanolic extract was mixed with distilled water + Folin–Ciocalteu’s reagent + Na_2_CO_3_ at room temperature. After 20 min, the absorbance spectrum was measured at 725 nm.

##### Nitric Oxide Content (NO)

Nitric oxide (NO) content was indirectly determined as described by Ding et al. [94] and modified by Hu et al. [95]. Briefly, NO was reacted with Greiss reagent (l% sulfanilamide/0.1% N-(l-naphthyl)-ethylenediamine dihydrochloride in 5% phosphate acid) was added for 30 min at room temperature till the appearance of azo violet color, which was measured spectrophotometrically at 550 nm. NaNO_2_ was used to prepare the standard curve and the NO content was expressed as nmol g^−1^ FW.

##### Phenylalanine Ammonia-Lyase (PAL) and Polyphenol Oxidase (PPO) Activities

Phenylalanine ammonia-lyase (PAL; EC 4.3.1.5) and Polyphenol oxidase (PPO; EC 1.10.3.1) activities were examined using the protocol of Havir and Hanson [96] and Kumar and Khan [97], respectively.

##### Flavonoids Content

Total flavonoid content was colorimetrically assessed as described by Zou et al. [98] using aluminum chloride with slight modifications. Briefly, 100 mg of leaf tissue was ground to a fine powder using liquid nitrogen, extracted with 1.0 mL methanol, then centrifuged at 10,000× *g* for 10 min. Subsequently, about 0.5 mL of the supernatant was mixed with 2 mL of distilled water and 150 µL of 5% sodium nitrate and incubated for 6 min. After incubation, 150 µL of aluminum chloride (10%) and 2 mL of sodium hydroxide (1 M) were added and re-incubated at room temperature for 15 min. The absorbance of the mixtures was measured at 510 nm. 

#### 4.6.5. Non-Enzymatic and Enzymatic Antioxidants

Non-enzymatic molecules in terms of reduced glutathione (GSH) and ascorbic acid (ASA) were studied based on the methods of Ellman [99] and Jagota and Dani [100]. The antioxidative enzymes of shoots were monitored by screening the specific activities of catalase (CAT, U mg^−1^ protein g^−1^ FW min^−^^1^), superoxide dismutase (SOD, U mg^−1^ protein g^−1^ FW min^−^^1^), ascorbate peroxidase (APX, µmol mg^−1^ protein g^−1^ FW min^−^^1^), guaiacol peroxidase (POD, (U mg^−1^ protein min^−^^1^) using the recommended procedures of Noctor et al. [101], Misra and Fridovich [102], Silva et al. [103] and Zaharieva et al. [104], respectively.

### 4.7. Statistical Analysis

Throughout this study, all experiments were laid out using a full factorial split-plot design arranged in completely randomized blocks using *R. solani*-infection (healthy vs. infected) as main plots and SiO_2_ treatments (0, 25, 50, and 100 µg mL^−1^ SiO_2_) in the subplots. All experiments were carried out using at least three biological replicates for each treatment. The analysis of variance (ANOVA) was used to test the significant differences among different infection levels (*p*_infection_), treatments (*p*_treatment_), and their interaction (*p*_infection×treatment_). Tukey’s honestly significant difference (HSD) test was used for post-hoc analysis (*p*_infection×treatment_ < 0.05). The data were analyzed using JMP data analysis software version 14.

## 5. Conclusions

In conclusion, SiO_2_ NPs treatment reduced the infection rate and suppressed the growth of *R. solani* on wheat. Our findings demonstrated that SiO_2_ NPs mitigate the negative effect of *R. solani* on wheat seedlings via the simultaneous activation of a multilayered defense system that involves at least three key mechanisms. (i) SiO_2_ NPs have a direct dose-dependent fungistatic activity against the vegetative growth of *R. solani.* (ii) SiO_2_ NPs induced the accumulation of defense-related compounds, particularly SA and its major biosynthetic enzyme PAL, and (iii) SiO_2_ NPs induce the activation of both enzymatic (POD, SOD, APX, CAT, and PPO) and non-enzymatic (phenolics and flavonoids) antioxidant defense machinery to neutralize the destructive effect of ROS, lipid peroxidation, and methylglyoxal to maintain their homeostasis within *R. solani*-infected plants. Collectively, it is plausible to propose that the application of SiO_2_ NPs could be considered as an alternative/eco-friendly approach to protect wheat plants from root rot-causing phytopathogenic fungi due to their suppressive role against the infection caused by *R. solani*.

## Data Availability

The data that supports the findings of this study are contained within the article or Supplementary Materials and available from the corresponding author upon reasonable request.

## Supplementary Materials

The following are available online at https://www.mdpi.com/article/10.3390/plants10122758/s1, Figure S1: Energy-dispersive X-ray spectroscopy (EDS) elemental mapping of Si K*α*1 (**A**) and O K*α*1 (**B**).
