# Supplementary material for: Silicon Dioxide Nanoparticles Induce Innate Immune Responses and Activate Antioxidant Machinery in Wheat Against Rhizoctonia solani
Source: Plants (Basel). 2021 Dec 14;10(12):2758. doi: 10.3390/plants10122758 (PMC8708575; doi:10.3390/plants10122758)
Supplement: Supplementary file 1 [file plants-10-02758-s001.zip › plants-1463909-supplementary.pdf]

Article

Biosynthesized Silicon Dioxide Nanoparticles Mitigate Wheat Damping-Off, Caused by *Rhizoctonia solani*, via the Simultaneous Activation of a Multilayered Defense System

Abdelrazek S. Abdelrhim [1], Yasser S. A. Mazrou [2,3], Yasser Nehela [4,5,*], Osama O. Atallah [6], Ranya M. El-Ashmony [1] and Mona F. A. Dawood [7]

1 Department of Plant Pathology, Faculty of Agriculture, Minia University, El-Minya 61512, Egypt; Abdelrazek.sharawy@mu.edu.eg (A.S.A); ranya.elashmoni@mu.edu.eg (R.M.E.-A.)
2 Business Administration Department, Community college, King Khalid University, Guraiger, Abha 62529, Saudi Arabia; ymazrou@kku.edu.sa
3 Department of Agriculture Economic, Faculty of Agriculture, Tanta University, Tanta 31527, Egypt; yasser.mazroua@agr.tanta.edu.eg
4 Department of Agricultural Botany, Faculty of Agriculture, Tanta University, Tanta 31511, Egypt; yasser.nehela@ufl.edu
5 Department of Plant Pathology, Citrus Research and Education Center, University of Florida, 700 Experiment Station Rd., Lake Alfred, FL 33850, USA
6 Department of Plant Pathology, Zagazig University, Zagazig 44519, Egypt; osamaoatall1h@ufl.edu
7 Botany and Microbiology Department, Faculty of Science, Assiut University, Assiut 71516, Egypt; mo_fa87@aun.edu.eg
* Correspondence: yasser.nehela@ufl.edu

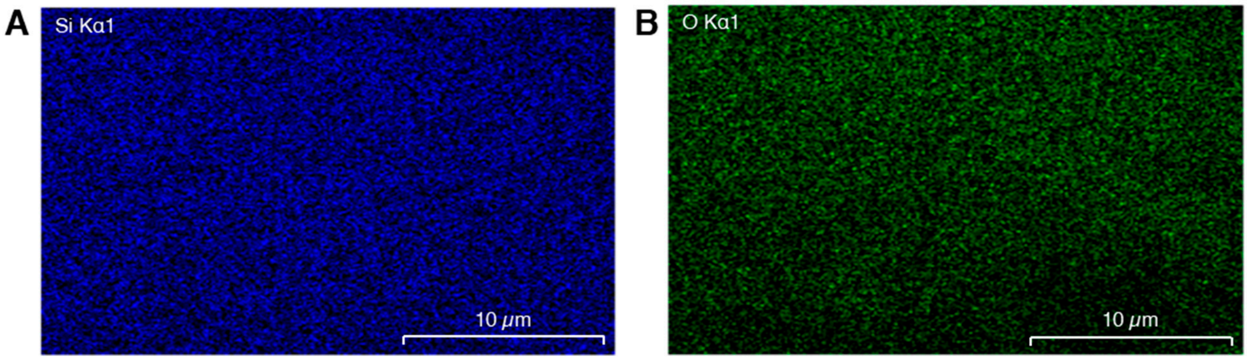

Figure S1. Energy-dispersive X-ray spectroscopy (EDS) elemental mapping of Si Kα1 **(A)** and O Kα1 **(B).**
